# Tranexamic acid in spontaneous intracerebral hemorrhage: an updated systematic review and meta-analysis of randomized controlled trials

**DOI:** 10.1080/07853890.2026.2635208

**Published:** 2026-03-03

**Authors:** Cuilin Que, Luda Feng, Xinxing Lai, Yonghong Gao, Hongrui Zhang, Chenxi Tao, Lingbo Kong, Zhenhong Liu, Ying Gao

**Affiliations:** a Department of Neurology, Dongzhimen Hospital, Beijing University of Chinese Medicine, Beijing, China; b Institute for Brain Disorders, Beijing University of Chinese Medicine, Beijing, China; c Dongfang Hospital, Beijing University of Chinese Medicine, Beijing, China; d Key Laboratory of Chinese Internal Medicine of Ministry of Education and Beijing, Dongzhimen Hospital, Beijing University of Chinese Medicine, Beijing, China

**Keywords:** Hematoma expansion, intracerebral hemorrhage, systematic review, tranexamic acid

## Abstract

**Background:**

Tranexamic acid (TXA) is a well-established antifibrinolytic medication in the general population. However, its efficacy and safety for patients with spontaneous intracerebral hemorrhage (ICH) remain inconclusive. Consequently, we conducted a systematic review and meta-analysis to assess the effectiveness and safety of TXA for spontaneous ICH.

**Methods:**

We conducted a systematic review and meta-analysis of randomized controlled trials (RCTs) following established methodological standards. Our search encompassed eight electronic databases from inception to April 25, 2024. The primary outcome was a reduction in all-cause mortality. The secondary outcomes included improvements in functional independence, neurological impairment, activities of daily living, and reduction in hematoma expansion (HE). Fixed-effects or random-effects model were performed for pooled data where eligible.

**Results:**

A total of 9 RCTs that initially enrolled 3,124 patients were included. There were no significant differences observed concerning all-cause mortality (RR, 1.03; 95% CI [0.89–1.18]), hematoma expansion (RR, 0.90; 95% CI [0.80–1.00]), improvement of functional independence (RR, 1.02; 95% CI [0.92–1.12], neurological impairment (MD, −0.88 [95% CI, −2.22–0.45]), or activities in daily living (MD, −0.83 [95% CI, −29.25–12.59]). The pooled data indicated that TXA for ICH was associated with a decrease in hematoma volume from baseline (MD, −1.74; 95% CI [−2.47 to −1.02]). No significant difference in adverse events was observed between the TXA group and the control group.

**Conclusions:**

In summary, TXA does not affect all-cause mortality, functional outcomes, or neurological impairment, nor does it reduce HE, despite reducing hematoma volulume. TXA use for ICH requires careful clinical consideration.

## Registration

URL: https://www.crd.york.ac.uk/PROSPERO/; Unique identifier: CRD42023467732.

## 1. Introduction

Spontaneous intracerebral hemorrhage (ICH) ranks as the second leading cause form of stroke, characterized by a significant mortality rate and persistent disability [1]. Consequently, it places a substantial economic impact on both patients’ families and society as a whole [2]. However, currently there are no approved medications for the treatment of ICH. Studies reveal that hematoma expansion (HE) is closely connected to poor prognosis, including early neurological deterioration, mortality [3]. HE is closely related to early neurological deterioration and poor prognosis in patients with ICH, which is a key target for the treatment of ICH. Therefore, seeking to inhibit early HE and lower the death rate among ICH patients is a crucial approach in the management of ICH.

Tranexamic acid (TXA) is a widely utilized antifibrinolytic medication in the management of bleeding patients [4]. Preclinical research has shown that TXA inhibits the HE *via* lowering blood-brain barrier permeability and having anti-inflammatory properties [5–7]. Despite the widespread administration of TXA to patients with ICH, its utilization profile remains a contentious issue as indicated by previous studies. Many systematic reviews show that TXA treatment can reduce the HE during spontaneous ICH [8–10]. However, two recent studies suggest that TXA is not related to hematoma volume [11,12]. Furthermore, TXA is not associated with a reduction in mortality. Hence, it is imperative to undertake a comprehensive meta-analysis and systematic review of TXA usage for patients having spontaneous ICH.

Accordingly, we performed this updated systematic review and meta-analysis to give a comprehensive evaluation of the efficacy and safety of TXA treatment among patients with ICH. The findings will provide the best current evidence regarding the use of TXA in ICH patients and offer valuable insights for clinical decision-making.

## 2. Methods

We registered the systematic review protocol in compliance with the Cochrane Handbook for Systematic Reviews of Interventions [13] and the reporting followed the Preferred Reporting Items for Systematic Reviews and Meta-analyses (PRISMA) guidelines [14]. This systematic review has been registered with the International Prospective Register of Systematic Reviews (CRD42023467732). Ethical approval was not required for this study because it simply used data from published research.

### 2.1. Search methodology and study selection

We searched for studies in great detail by PubMed, Web of Science, Embase, Cochrane Library, China National Knowledge Infrastructure, Chinese VIP information, Wanfang Data, and SinoMed from inception until April 25, 2024. Supplement 1 contains the comprehensive search tactics (Table S1). Additionally, we conducted a language-neutral search of dissertations, registered clinical trials, current or unpublished research, and gray literature. In addition to the database search, a manual review of the reference lists from the included studies and previous systematic reviews was conducted. After eliminating duplicate records from the retrieved studies, two reviewers (Cuilin Que and Luda Feng) independently examined the articles by titles, abstracts, and full texts to assess their eligibility. All discrepancies were settled through discussion and, if needed, by contacting a senior author (Ying Gao).

### 2.2. Inclusion and exclusion criteria

Studies that satisfied the following requirements were deemed eligible: (1) the participants were patients with spontaneous ICH; (2) adult patients with spontaneous ICH (≥18 years old); (3) TXA or TXA with conventional treatments was the primary intervention examined; (4) the study had clinical control groups, consisting of either no treatment, placebo, or conventional treatments when administered in both groups; and (5) studies must have reported on at least just one of the following outcomes: all-cause mortality among the ICH population, improvement of functional independence, neurological impairment, activities of daily living and HE; (6) only RCT studies were included. There were no limitations on the patients’ sex, severity of spontaneous ICH or method of medication delivery. Moreover, studies that enrolled subjects who suffered from subarachnoid hemorrhaging, spontaneous intraventricular hemorrhaging, or traumatic hemorrhage were not included.

### 2.3. Data extraction

Data extraction was carried out by four reviewers (Cuilin Que, Luda Feng, Hongrui Zhang and Zhenhong Liu) in pairs using a predefined extraction form, With any inconsistencies settled through conversation. If no consensus could be obtained, a senior author (Ying Gao) made the final decision. The primary outcome was all-cause mortality. The secondary outcomes of interest included functional independence, defined as a modified Rankin scale (mRS) grade; neurological impairment according to the National Institutes of Health Stroke Scale (NIHSS) score; activities in daily living measured with the Barthel Index (BI); hematoma volume change; and HE. Safety events included thromboembolic events and nervous system disorders.

### 2.4. Risk-of-bias evaluation and evidence certainty

Reviewers (including Cuilin Que and Chenxi Tao) used the Cochrane Risk of Bias Tool 2.0 and evaluated each study’s risk of bias separately, taking into account all possible outcomes. The following domains were included in this risk-of-bias tool: randomization process, deviations from intended interventions, missing outcome data, measurement of the outcome, selection of the reported result, and overall bias, among more biases. The evidence supporting the primary outcomes was divided into four groups using the Grading of Recommendations Assessment, Development, and Evaluation (GRADE) technique: high, moderate, low, and very low [15]. Two reviewers (Luda Feng and Zhenhong Liu) utilized the GRADE approach to evaluate the degree of certainty in the evidence with respect to the risk of publication bias, indirectness, imprecision, bias, and inconsistency [16–22]. A senior member of the research team reconciled any differences (Ying Gao).

### 2.5. Analysis of statistics

We used RevMan 5.3 and STATA 14.0 software for all the statistical analyses. To compile the data for binary outcomes (all-cause mortality, functional independence, and adverse events), we calculated risk ratios (RR) and 95% confidence intervals (CIs), and we used the mean difference (MD) with 95% CI to assess the credibility of the estimates (neurological deficits, activities of daily living, and HE). Statistical significance was determined as a two-sided P-value less than 0.05. The degree of statistical heterogeneity across the included studies was assessed using the *I*^2^ test. When *I^2^* was less than 50%, the pooled effect size was calculated using the fixed effect model. When *I^2^* exceeded 50%, the random effect or qualitative analysis model was used. Subgroup analyses by visit time and dose range of TXA were conducted. We performed a leave-one-out sensitivity analysis, calculating the pooled impact estimates for the studies that remained after eliminating each trial one at a time, to examine the impact of every investigation on the total effect size. Considering the clinical heterogeneity, we employed the random effects model to assess the stability of the findings. Preference is given to the random effects model’s outcomes when the fixed effects model’s and the random effects model’s conclusions are inconsistent [23].

## 3. Results

### 3.1. Selection of studies

**Figure 1.**
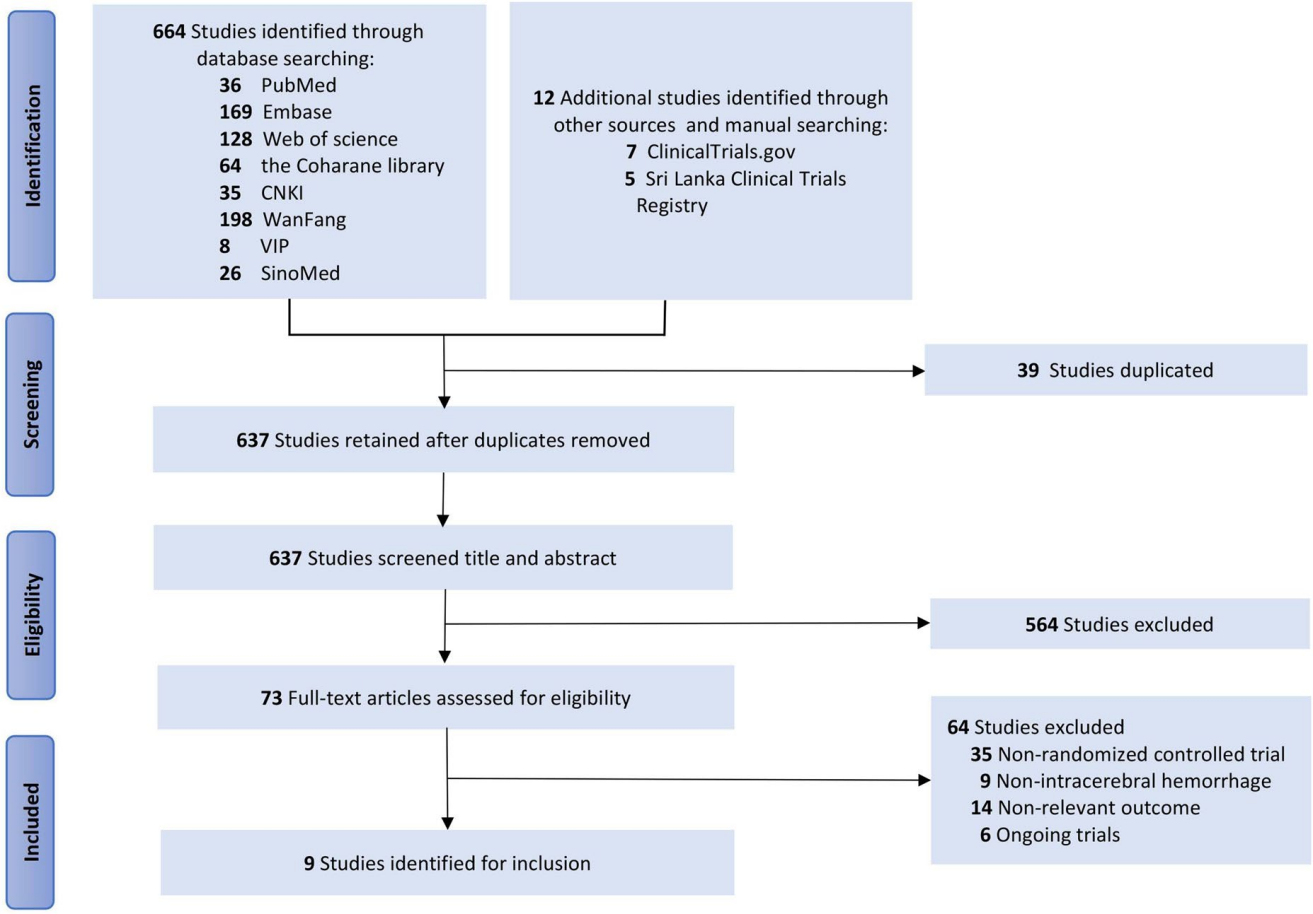
Flow diagram of identification, screening, and evaluation process.

The methodical search of the digital repositories and clinical trials registry platform initially yielded 676 records, and after removing duplicates, 637 unique records remained. After the assessment of 637 titles, abstracts, and 73 complete articles, we ultimately included 9 RCTs [11,12,24–30]. One of these investigations was conducted in Chinese, and the others were conducted in English. The PRISMA flow diagram represents the study selection approach (Figure 1).

### 3.2. Study characteristics

Overall, we included 9 relevant RCTs that included an overall amount of 3,124 patients (57% male) with spontaneous ICH, of whom 1,579 received TXA treatment. There were 4 single-center studies [24,26–28] and 5 multicenter studies [11,12,25,29,30]. The RCTs, published between 2014 and 2024, were conducted in 17 countries across Asia, Europe, North America, and Oceania. The mean (SD) age range was 51.6 (11.73) to 82 (8.89) years. The sample size ranged from 30 to 2,325 participants. The mean (SD) GCS score arrayed from 10.33 (1.80) to 15 (1.48), while the onset of ICH symptoms occurred within 24 h. All nine included RCTs [11,12,24–30] compared TXA against placebo. Eight studies [11,12,25–30] evaluated TXA monotherapy, while one trial [30] assessed TXA combined with conventional therapy. Although all studies were placebo-controlled, one [24] employed an open-label design. Table 1 presents an overview of the study participants’ baseline characteristics.

**Table 1.** Characteristics of the 9 eligible studies.

Source	Year	Study site	Sample size, no	Gender (male/female), no		Age mean (SD), years		Glasgow Coma Scale (GCS) mean (SD), score		Symptom of onset	ICH location and sample, no	Outcomes
				*T*	*C*	*T*	*C*	*T*	*C*			
Ni [24]	2020	Single center	73⁄77	40/33	43/34	69.11(8.45)	68.72(8.41)	10.33(1.80)	10.86(1.94)	<8h^a^	· Basal ganglia 82 · Cerebral lobe 46 · Cerebellum 21 · Brain stem 1	①②③④⑤⑥
STOP-AUST [25]	2020	Multicenter	50/50	35/15	27/23	73 (17.04)	71(15.56)	14 (2.96)	15 (1.48)	<4.5h	· Pure cerebellar 1 · Hemispheric cortical 30 · Hemispheric deep 69 · Mixed 0	①②⑤⑥
STOP-MSU [12]	2024	Multicenter	103/98	63/40	56/42	65.0(16.3)	67.0(14.81)	15 (1.48)	15 (0.74)	<2h	· Hemispheric cortical 33 · Hemispheric deep 168 · Cerebellar 0	①②⑤⑥
TANICH I [26]	2015	Single center	15/15	18/12		52.93		14.3 (2.67)		<8h	· Caudate 2 · Thalamus 4 · Internal capsule 24 · Right hemisphere 23 · Left hemisphere 7	①⑤⑥
TANICH II [27]	2023	Single center	20/20	24/16	12/8	51.6(11.73)	52.09(10.05)	NR^b^	NR	<8h	· Putamen 47 · Thalamus 8 · Subcortex 2 · Other 3	①②⑤⑥
TICH-1 [28]	2014	Single center	16/8	10/6	4/4	67.9 (13.2)	68.5 (12.9)	12.7 (3.1)	12.8 (2.7)	<24h	· Thalamus 10 · Basal ganglia 6 · Cerebral lobe 8	①④⑤⑥
TICH-2 [29]	2018	Multicenter	1161/1164	642/519	659/505	69.1 (13.7)	68.7 (13.9)	13 (2.2)	14 (2.1)	<8h	· Supratentorial lobar 738 · Supratentorial deep 1371 · Infratentorial 149 · Combination 67	①②③④⑤⑥
TICH-NOAC [11]	2023	Multicenter	32/31	18/14	20/11	82 (8.89)	81 (5.93)	13.5(1.48)	14 (2.22)	<12h	· Nonlobar 44 · Lobar 19	①②⑤⑥
TRAIGE [30]	2021	Multicenter	89/82	63/26	61/21	56.7 (12.2)	55 (10.8)	14 (2.96)	14 (2.96)	<6h	· Supratentorial lobar 44 · Supratentorial deep 127 · Thalamus 16 · Basal ganglia 111	①②③⑤⑥

^a^ h, hour; ^b^NR, not reported; ① All-cause Mortality; ② Functional Independence; ③ Neurological Impairment Score; ④ Activities of Daily Living; ⑤ Hematoma Volume Change and Hematoma Expansion; ⑥ Adverse Events.

**Figure 2.**
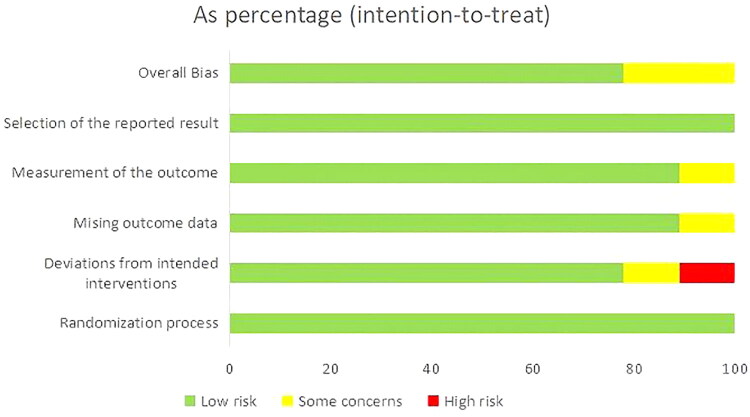
Risk of bias graph. A low risk of bias is shown by green plus signs, an unclear risk of bias is represented by yellow question marks, and a high risk of prejudice is indicated by red minus signs.

Figure 2 and Figure S1 provide summaries of each individual and aggregate study-level quality bias, respectively. The results indicated that one of the nine RCTs had a strong potential for prejudice because of the investigator’s knowledge of the study allocation.

### 3.3. Primary outcomes

#### 3.3.1. All-cause mortality

**Figure 3.**
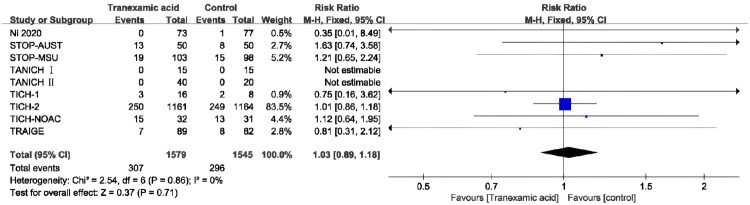
Forest plot of the all-cause mortality.

Nine RCTs [11,12,24–30] reported the all-cause mortality. The low level of heterogeneity led to the selection of a fixed effect model (*p* = 0.86; *I*^2^ = 0%). According to the combined results, the TXA group’s all-cause mortality was not significantly different from the control group’s (RR, 1.03; 95% CI [0.89–1.18]; *p* = 0.71) (Figure 3).

### 3.4. Secondary outcomes

#### 3.4.1. Functional independence

**Figure 4.**
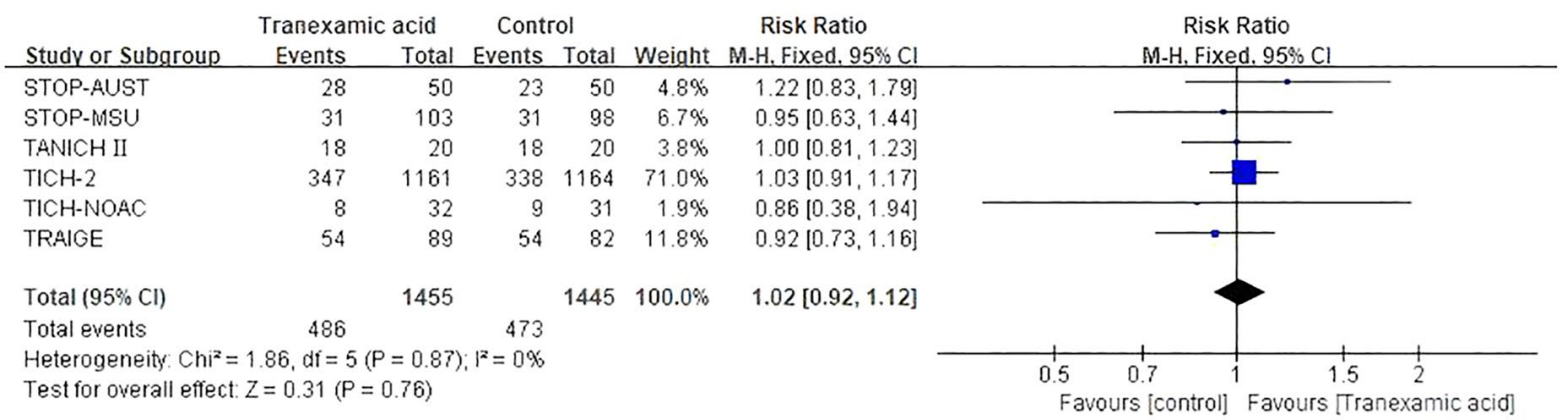
Forest plot of the modified Rankin Scale score of 0 to 3.

Six RCTs [11,12,25,27,29,30] which included 2,900 patients, reported mRS scores. A fixed effect model was chosen because of the low heterogeneity (*p* = 0.87; *I*^2^ = 0%). The combined data demonstrated that the addition of TXA did not lower the mRS score when compared to those in the control group (RR, 1.02; 95% CI [0.92 to 1.12]; *p* = 0.76) (Figure 4). Subgroup analyses were also performed, dividing the patients into two subgroups based on visit time (30-day visit: MD, 1.00; 95% CI [0.81–1.23]; *p* = 1.00; 90-day visit: MD, 1.02; 95% CI [0.92–1.12]; *p* = 0.87; *I*^2^ = 0%) (Figure S2). The results indicated that the administration of TXA did not result in a reduction of the mRS score on Day 30 or Day 90.

#### 3.4.2. Neurological impairment score

**Figure 5.**
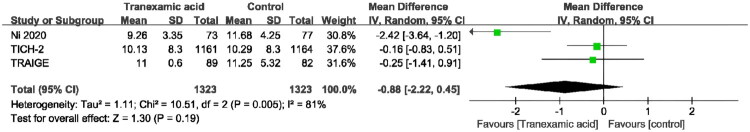
Forest plot of the effect of tranexamic acid on neurological impairment score.

Three RCTs [24,29,30] which included 2,646 patients, reported on the NIHSS. The meta-analysis results showed that TXA did not lower the NIHSS score (MD = −0.88; 95% CI [−2.22 to 0.45]; *p* = 0.19) (Figure 5). However, there was significant heterogeneity among studies (*I*^2^ = 81%; *p* = 0.005). A random effects model was chosen for the study. According to the sensitivity analysis eliminating the outlier RCTs [30], the heterogeneity of the remaining RCTs decreased. However, TXA did not significantly reduce the NIHSS score (MD, −0.18; 95% CI [−0.77 to 0.40]; *p* = 0.54) (Figure S3).

#### 3.4.3. Improvement in activities of daily living

**Figure 6.**
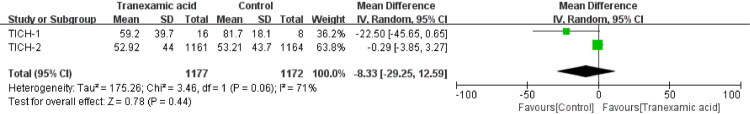
Forest plot of the effect of tranexamic acid on Barthel index.

Two RCTs [28,29] reported grading based on the Barthel Index (BI). The great heterogeneity led to the selection of a random effect model (*I*^2^ = 71%, *p* = 0.06). However, these RCTs did not provide any evidence to support the improvement of BI with TXA (MD, −8.33; 95% CI [−29.25–12.59]; *p* = 0.44) (Figure 6).

#### 3.4.4. Hematoma volume change and hematoma expansion

**Figure 7.**
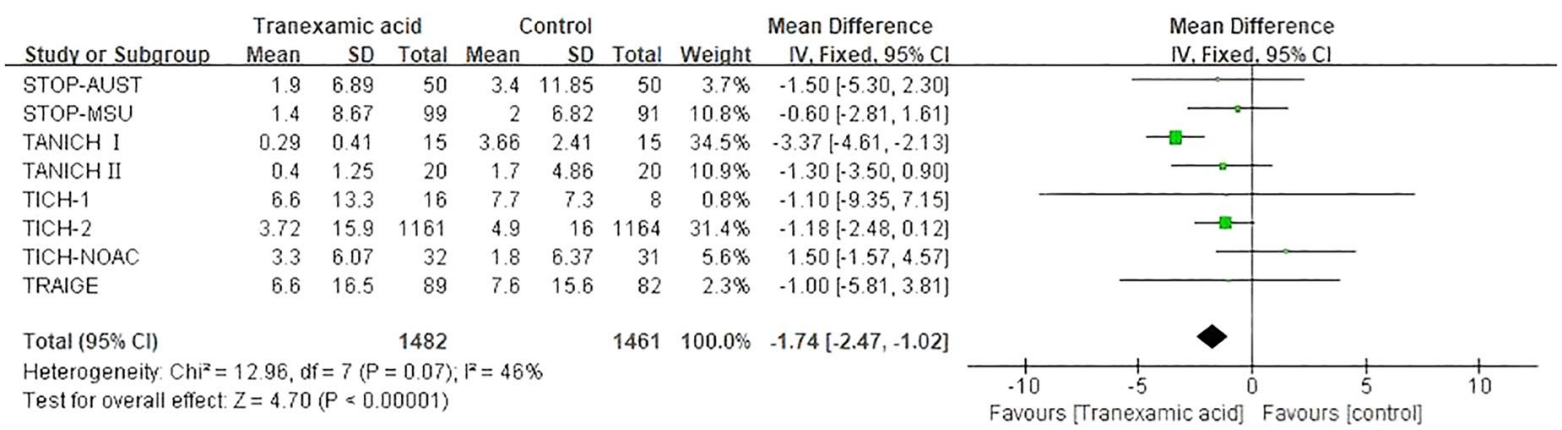
Forest plot of tranexamic acid on the increased hematoma volume.

Eight articles [11,12,25–30] reported elevated hematoma volume. Notably, compared with placebo, TXA significantly reduces the change in hematoma volume from baseline (MD, −1.74; 95% CI [−2.47 to −1.02]; *p* < 0.00001; *I*^2^ = 46%) (Figure 7). Subgroup analyses were also performed, dividing the patients into two subgroups based on severity of GCS (GCS 13–15, MD, −1.31; 95% CI [−2.76 to 0.15]; *p* = 0.08; *I*^2^ = 61%; GCS 9–12, MD, −1.10; 95% CI (−9.35 to 7.15); *p* = 0.79]) (Figure S4).

**Figure 8.**
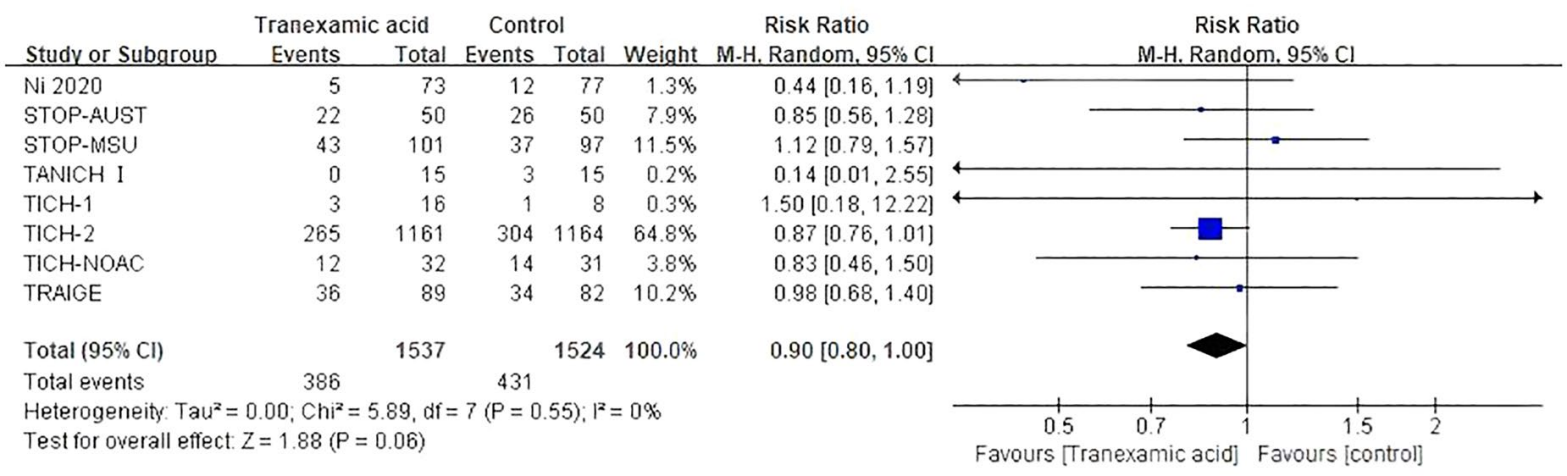
Forest plot of tranexamic acid on hematoma expansion.

Additionally, eight articles [11,12,24,26–30] reported HE. We first used a fixed effects model (*I*^2^ = 0%, *p* = 0.55). The combined data showed that HE was slightly lower in the TXA group than in the control group (RR, 0.88; 95% CI [0.79**–**0.99]; *p* = 0.04) (Figure S5). To make the results more credible, a random effects model was used to analyze the data. Interestingly, the results indicated that TXA seemed to be unable to reduce HE (RR, 0.90; 95% CI [0.80–1.00]; *p* = 0.06) (Figure 8).

#### 3.4.5. Adverse events

**Figure 9.**
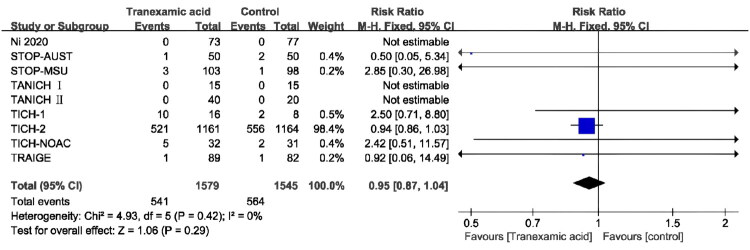
Forest plot of the adverse events.

A total of nine articles [11,12,24–30] reported adverse events. Of these, the nine RCTs reported no statistically significant difference in adverse events between the TXA and placebo groups (RR, 0.95; 95% CI [0.87**–**1.04]; *p* = 0.29; *I*^2^ = 0%) (Figure 9). During or after TXA treatment, a cumulative total of 541 participants experienced adverse events, with thromboembolic events and nervous system disorders being the most frequently reported. We analyzed the occurrence of thromboembolic events (RR, 1.16; 95% CI [0.76**–**1.75]; *p* = 0.49; *I*^2^ = 0%) (Figure S6) and nervous system disorders (RR, 0.91; 95% CI [0.74**–**1.12]; *p* = 0.37; *I*^2^ = 0%) (Figure S7).

### 3.5. Certainty of the evidence

The certainty regarding the effect of TXA on the BI score was classified as ‘low.’ Similarly, for outcomes such as all-cause mortality, the mRS score, the NIHSS score, and adverse events were rated as ‘moderate’, and for hematoma volume change and hematoma expansion, they were rated as ‘high’ (Table 2). We determined that the quality of the evidence was moderate mostly because of the confidence interval intersecting with the null line and not being statistically significant, as well as serious inconsistency.

**Table 2.** GRADE evidence profiles.

Outcomes	No. of participants (no. of studies)	Certainty assessment					Relative effect (95% CI)	Absolute effect (95% CI)	Certainty
		Risk of bias	Inconsistency	Indirectness	Imprecision	Other considerations			
All-cause mortality	3,124(9)	Serious	Not serious	Not serious	Serious^a^	None	RR 1.03 (0.89–1.18)	–	⨁⨁⨁◯ Moderate
mRS	2,900(6)	Serious	Not serious	Not serious	Serious^a^	None	RR 1.02 (0.92–1.12)	–	⨁⨁◯◯ Low
NIHSS	2,646(3)	Serious	Serious^b^	Not serious	Not serious	None	–	MD −0.88 (−2.22 to 0.45)	⨁⨁⨁◯ Moderate
BI	2,349(2)	Serious	Serious^b^	Not serious	Serious^a^	None	–	MD −8.33 (−29.25 to 12.59)	⨁⨁◯◯ Low
Hematoma volume change	2,943(8)	Not serious	Not serious	Not serious	Not serious	None	–	MD −1.74 (−2.47 to −1.02)	⨁⨁⨁⨁ High
HE	3,061(8)	Not serious	Not serious	Not serious	Not serious	None	RR 0.90 (0.80–1.00)	–	⨁⨁⨁⨁ High
Adverse events	3,124(9)	Not serious	Not serious	Not serious	Serious^a^	None	RR 0.95 (0.87–1.04)	–	⨁⨁⨁◯ Moderate

Explanations.

^a^ The confidence interval intersects the null line and is not statistically significant.

^b^ Serious inconsistence.

## 4. Discussion

The findings of our study suggest that TXA had no impact on all-cause mortality, nor did it increase the probability of good long-term functional outcomes or improvements in neurological impairment. It is worth mentioning that our results showed that TXA was ineffective in reducing HE, which contradicts the findings of previous systematic reviews, although it did reduce hematoma volume. Additionally, TXA did not improve outcomes, according to our subgroup analysis based on the time of visit for mRS. Notably, the incidence of adverse events was similar in both groups.

The fixed-effect and random-effect models were used in the HE analysis. The fixed-effect model results indicated that TXA may slightly reduce the incidence of HE, whereas the random-effect model results showed the opposite trend. Considering the studies in this meta-analysis were not conducted on the same population, and that the encompassed RCT studies were being made in 17 countries with varying durations of intervention ranging from 2 to 24 h, thus making the random-effects results more conservative. In addition, it has been suggested that the random effects model would be a more appropriate choice in most meta-analyses [23]. However, the results of the fixed effect model indicated that TXA appeared to be beneficial in reducing HE. This is probably because the TICH-2 study with 2,325 patients showed that TXA was effective in reducing HE, which may lead to an overestimation of the pooled results in our systematic review. The primary highlight of our meta-analysis revealed that TXA did not demonstrate significant efficacy in reducing HE. We postulated that this outcome may be attributed to recent findings from TICH-NOAC [11] and STOP-MSU [12], which indicated that TXA was ineffective in reducing HE and therefore could not be recommended for clinical application.

TXA’s safety record for patients receiving treatment for ICH remains uncertain, based on earlier meta-analyses [31]. We found no statistically significant variation in safety outcomes from our meta-analysis of RCTs. This study demonstrated that thromboembolic events and nervous system disorders were the main adverse events. However, no notable differences were found between the groups.

The choice of medication and intervention may vary based on the bleeding source, potentially leading to better outcomes Theoretically, the significance of antifibrinolytic and hemostatic medicines in the treatment of ICH has led to a resurgence of interest in these modalities [32]. The application of these treatment methods might successfully lessen the degree of bleeding in individuals with ICH, improve patient quality of life, increase the survival rate, and lower the frequency of complications. The effectiveness of TXA in treating patients with spontaneous ICH remains controversial in the literature. TXA is an antifibrinolytic drug that has attracted the attention of researchers in the management of ICH in the past few years [33]. Several studies have indicated that TXA may decrease bleeding and enhance patient prognosis by inhibiting plasmin activity and reducing vascular permeability at the site of hemorrhage [34]. Furthermore, TXA might be involved in protecting nerve cells by inhibiting the inflammatory response and reducing apoptosis [35].

In previous studies [8–10,36–38] only hematoma volume expansion, mortality, and adverse events were described. These reviews did not reflect the scores of activities of daily living or the degree of neurological deficit in terms of outcome measures. We summarized three previously published meta-analyses. One of the meta-analyses indicated that the treatment of adult spontaneous ICH with TXA, compared to either a placebo or no medical intervention, can reduce the incidence of ICH [36]. Another meta-analysis conducted a thorough investigation, and its findings suggested that TXA can reduce the possibility of HE in individuals suffering from acute spontaneous ICH. Treatment within 4.5 h can lower the risk for individuals who have a high risk of hematoma growth, while treatment beyond 4.5 h does not lead to a reduction in risk [10]. However, another study [39] focused on the deaths and thromboembolic events associated with TXA use in trauma patients. The results point to the potential benefits of TXA for a number of traumatized patients. Nonetheless, legitimate worries over the potential of TXA to cause thromboembolic events persist. Several related studies are shown in Table 3.

**Table 3.** Comparisons of several studies.

Meta-analysis	Guo 2021 [8]	Yu 2021 [9]	Wang 2021 [10]	Que 2026
Study population	Spontaneous ICH	Spontaneous ICH	Spontaneous ICH	Spontaneous ICH
Number of included studies	7 RCTs	4 RCTs and 1 retrospective case series	6 RCTs	9 RCTs
All-cause mortality	OR = 1.02, 95% CI: 0.843–1.234; *p* = 0.834	OR = 1.14, 95% CI: 0.73–1.77; *p* = 0.58	RR = 1.02, 95% CI: 0.88–1.19; *p* = 0.80	RR = 1.03, 95% CI: 0.89–1.18; *p =* 0.71
Long-term functional outcomes	NA	mRS ≤ 3: OR = 1.06, 95% CI: 0.89–1.26; p = 0.51	NA	mRS ≤ 3: RR = 1.02, 95% CI: 0.92–1.12; *p* = 0.76
Improvement of neurological impairment	NA	NA	NA	NIHSS: MD = –0.8, 95% CI: −2.22 to 0.45; *p* = 0.19
Improvement of activities of daily living	NA	NA	NA	BI: MD = –8.33, 95% CI: −29.25 to 12.59; *p* = 0.44
Hematoma volume change	NA	MD = –1.06, 95% CI: −2.02 to 0.09; *p* = 0.03	MD = –1.28, 95% CI: −2.44 to − 0.12; p = 0.03	MD = –1.74, 95% CI: −2.47 to −1.02; *p* < 0.00001
HE	OR = 0.825, 95% CI: 0.692–0.984; *p* = 0.033	OR = 0.81, 95% CI: 0.68–0.97; *p* = 0.02	RR = 0.87, 95% CI: 0.77–0.99; p = 0.03	RR = 0.90, 95% CI: 0.80–1.00; *p* = 0.06
Adverse events	NA	Major thromboembolic events: OR = 1.04, 95% CI:0.67–1.62; p = 0.87	Major thromboembolic complications: RR = 1.20, 95% CI: 0.85–1.69; p = 0.80	(1) all adverse events: RR = 0.95 95% CI: 0.87–1.04; *p* = 0.29; (2) thromboembolic events: RR = 1.16, 95% CI: 0.76–1.75; *p =* 0.49; (3) nervous system disorders: RR = 0.91, 95% CI: 0.74–1.12; *p =* 0.37
Clinical recommendations	Favourable	Favourable	Favourable	Neutral

We included only patients with spontaneous ICH in our investigation, and we discovered that in contrast to a placebo, TXA treatment can reduce HE in these patients. It should be stressed that this meta-analysis is the first to concentrate on patients with spontaneous ICH because of improvements in neurological impairment, long-term functional outcomes, and daily living activities in comparison to earlier studies. We aimed to investigate the efficacy of TXA in patients with spontaneous ICH more comprehensively and objectively.

Initially, the majority of studies within this domain were endeavored to gather, consequently furnishing the most thorough evidence of TXA’s participation in ICH. There may be some potential limitations to our investigation. First, the inclusion of only 9 RCTs with relatively small sample sizes may limit the generalizability of the results and could affect the stability of the results. Additionally, we included open-label trials, resulting in low evidence quality. However, the quality of evidence in most RCTs is moderate-to-high, which ensures the power of the research conclusions. Finally, due to the lack of data, a more in-depth subgroup analysis to observe the impact of different time points of drug administration on HE in acute spontaneous ICH was not conducted, hence preventing the determination of the optimal timing for TXA administration. Future work should consider incorporating imaging and molecular biomarkers to investigate interactive effects, and propose individualized treatment hypotheses for specific subgroups.

## 5. Conclusions

In brief, we maintain a neutral attitude towards the use of TXA for ICH in clinical decision-making. TXA did not reduce HE or have an impact on all-cause mortality, nor did it increase the probability of good long-term functional outcomes or improvements in neurological impairment, despite its ability to reduce hematoma volume. It remains unclear whether TXA can effectively treat ICH, and high-quality trials at the patient level are warranted.

## Supplementary Material

Supplemental Material

Supplemental Material

Fig S1.jpg

Fig S3.jpg

Fig S5.jpg

Table S1.docx

Fig S4.jpg

Fig S7.jpg

Fig S6.jpg

PRISMA checklist.docx

## Data Availability

The data that support the findings of this study are available from the corresponding author, Ying Gao and Zhenhong Liu, upon reasonable request.

## Preprint server

This manuscript has not been posted to any community preprint server.

## Abbreviations

BI: Barthel Index
BBB: Blood–brain barrier
CI: Confidence interval
GRADE: Grading of Recommendations Assessment, Development, and Evaluation
HE: Hematoma expansion
ICH: Intracerebral hemorrhage
MD: Mean difference
mRS: modified Rankin Scale
NIHSS: National Institute of Health Stroke Scale
PRISMA: Preferred Reporting Items for Systematic Reviews and Meta-analyses
RCTs: Randomized clinical trials
RR: Risk ratio
TXA: Tranexamic acid
