# Supplementary material for: Tranexamic acid in spontaneous intracerebral hemorrhage: an updated systematic review and meta-analysis of randomized controlled trials
Source: Ann Med. 2026 Mar 3;58(1):2635208. doi: 10.1080/07853890.2026.2635208 (PMC12961703; doi:10.1080/07853890.2026.2635208)
Supplement: Supplemental Material [file IANN_A_2635208_SM4152.docx]

**Supplemental Figure caption**

Fig. S1 Risk of bias of each RCTs.

Fig. S2 Forest plot of subgroup analysis of visit time on mRS.

Fig. S3 Forest plot of sensitivity analysis of the NIHSS.

Fig. S4 Forest plot of subgroup analysis of severity of GCS of hematoma expansion.

Fig. S5 Forest plot of tranexamic acid on hematoma expansion (fixed effects model)

Fig. S6 Forest plot of thromboembolic events. 24 Fig. S7 Forest plot of neurological deterioration events.
