# Supplementary figures and images for: Tranexamic acid in spontaneous intracerebral hemorrhage: an updated systematic review and meta-analysis of randomized controlled trials
Source: Ann Med. 2026 Mar 3;58(1):2635208. doi: 10.1080/07853890.2026.2635208 (PMC12961703; doi:10.1080/07853890.2026.2635208)

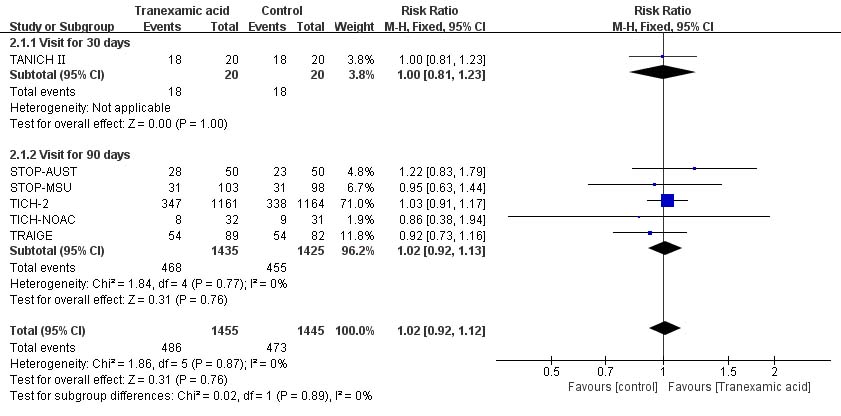

Supplement: Supplemental Material [file IANN_A_2635208_SM3815.jpg]

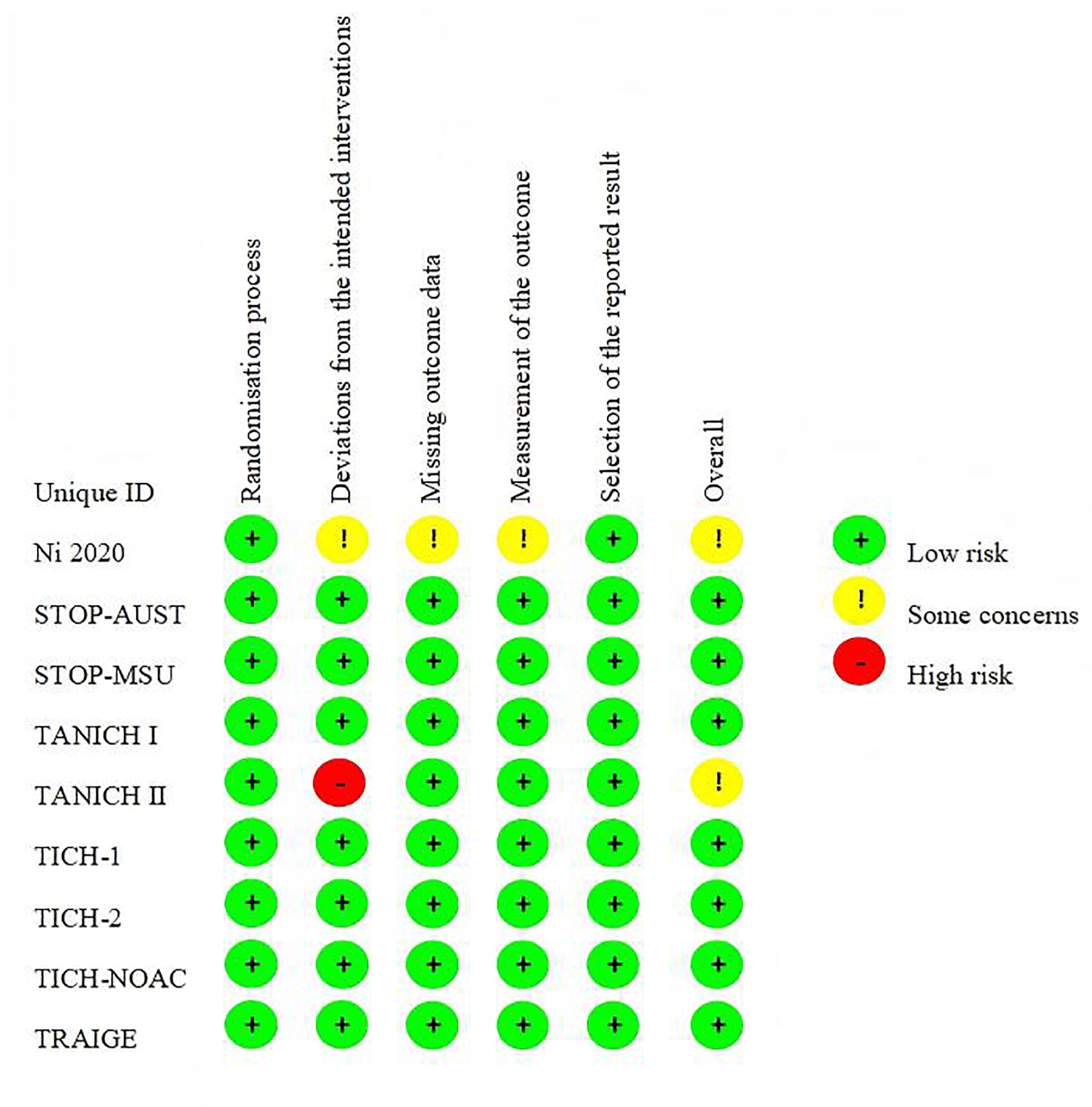

Supplement: Fig S1.jpg [file IANN_A_2635208_SM2013.jpg]

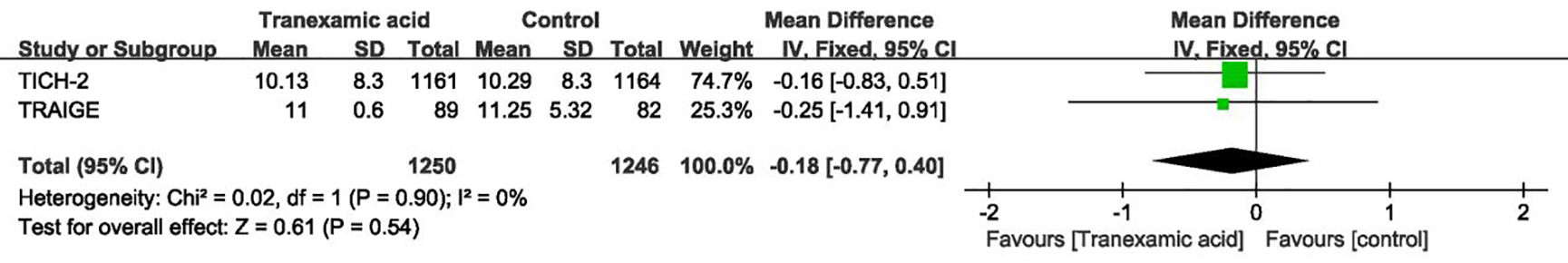

Supplement: Fig S3.jpg [file IANN_A_2635208_SM2012.jpg]

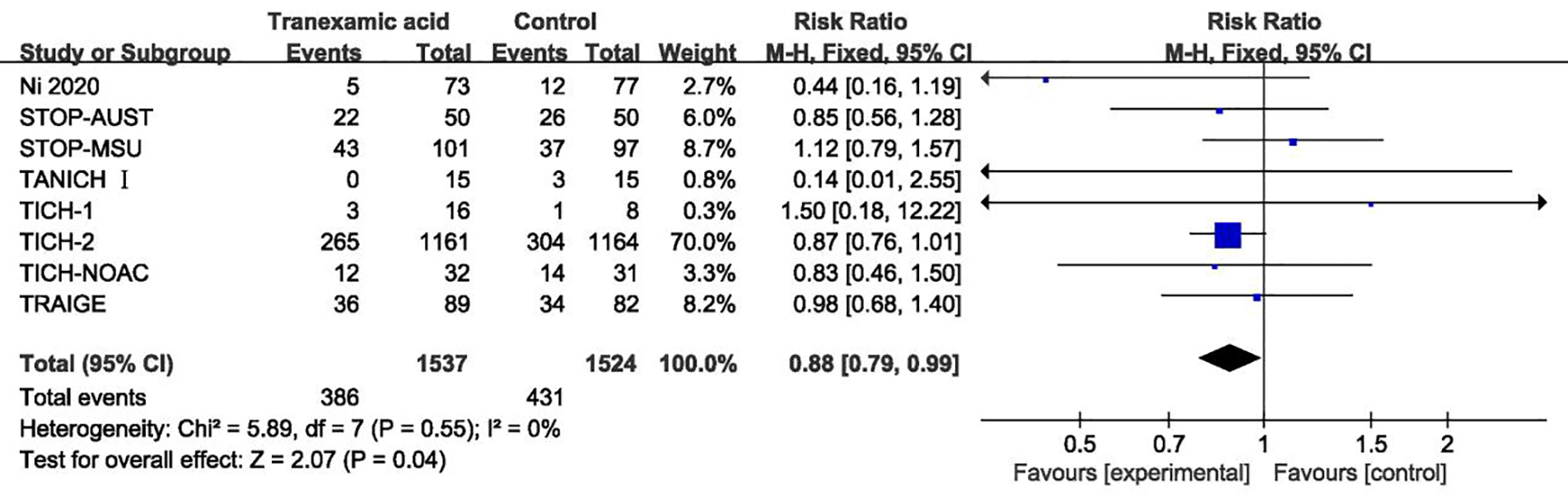

Supplement: Fig S5.jpg [file IANN_A_2635208_SM2011.jpg]

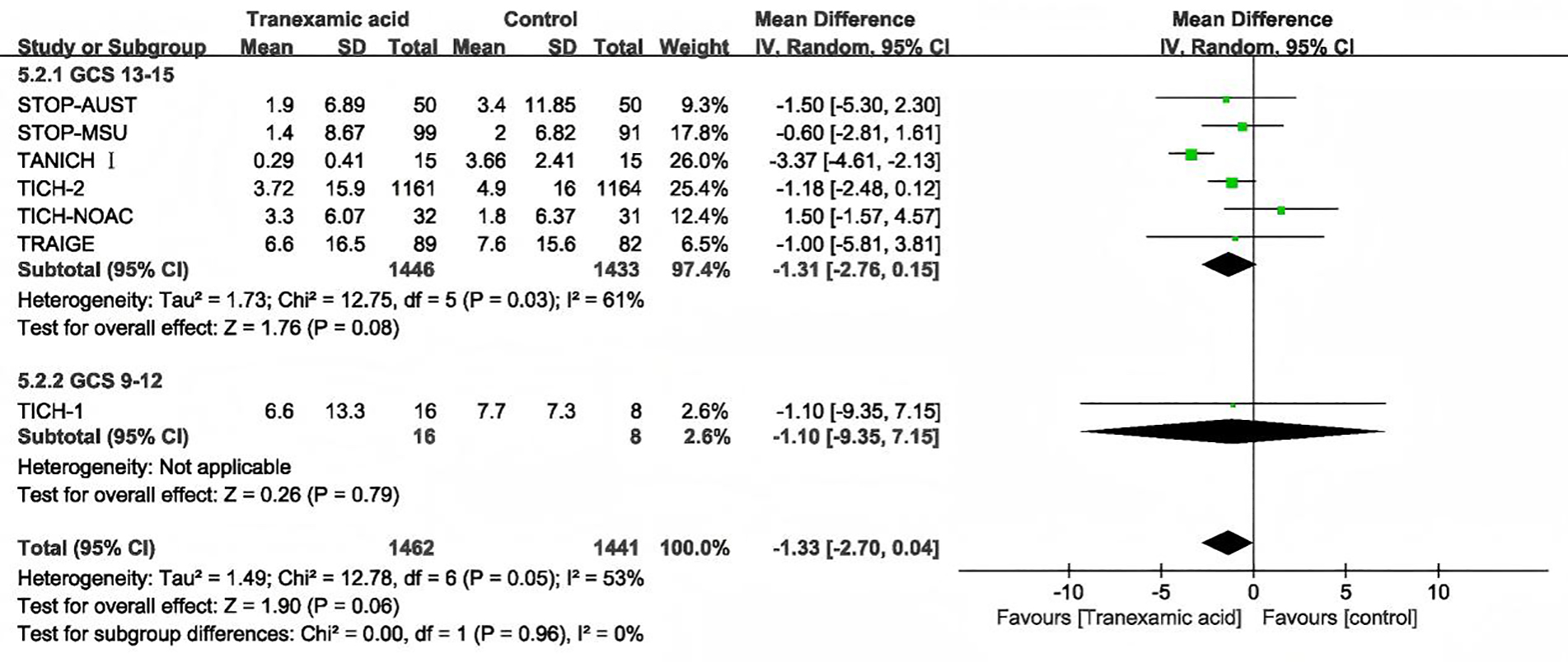

Supplement: Fig S4.jpg [file IANN_A_2635208_SM2009.jpg]

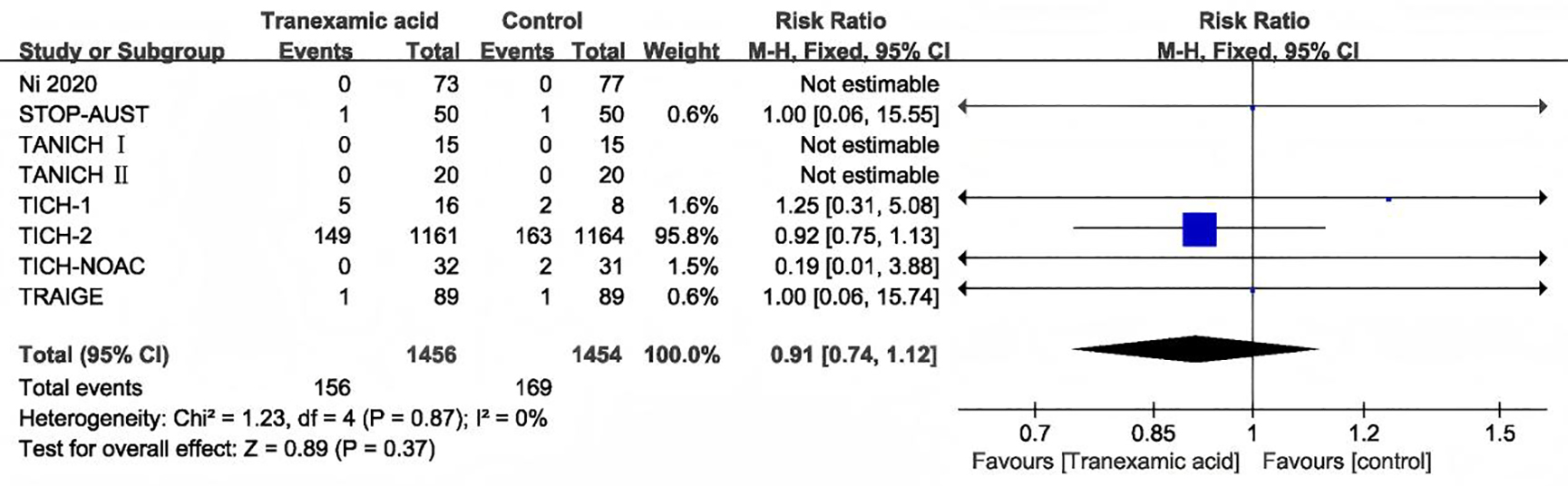

Supplement: Fig S7.jpg [file IANN_A_2635208_SM2008.jpg]

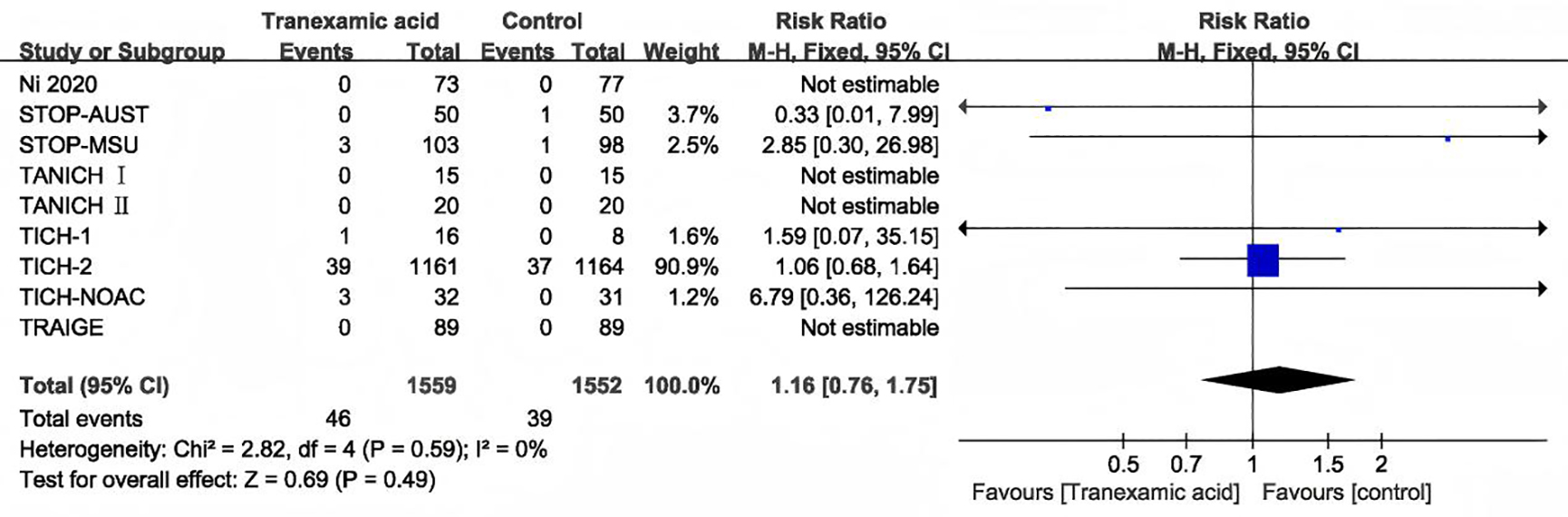

Supplement: Fig S6.jpg [file IANN_A_2635208_SM2007.jpg]
