# Supplementary material for: Tranexamic acid in spontaneous intracerebral hemorrhage: an updated systematic review and meta-analysis of randomized controlled trials
Source: Ann Med. 2026 Mar 3;58(1):2635208. doi: 10.1080/07853890.2026.2635208 (PMC12961703; doi:10.1080/07853890.2026.2635208)
Supplement: Table S1.docx [file IANN_A_2635208_SM2010.docx]

Table S1. Search strategy example: PubMed search.

| **No** | **Search items** |
| --- | --- |
| 1 | Cerebral Hemorrhage |
| 2 | Hemorrhage, Cerebrum |
| 3 | Cerebrum Hemorrhage |
| 4 | Cerebrum Hemorrhages |
| 5 | Hemorrhages, Cerebrum |
| 6 | Cerebral Parenchymal Hemorrhage |
| 7 | Cerebral Parenchymal Hemorrhages |
| 8 | Hemorrhage, Cerebral Parenchymal |
| 9 | Hemorrhages, Cerebral Parenchymal |
| 10 | Parenchymal Hemorrhage, Cerebral |
| 11 | Parenchymal Hemorrhages, Cerebral |
| 12 | Intracerebral Hemorrhage |
| 13 | Intracerebral Haemorrhage |
| 14 | Hemorrhage, Intracerebral |
| 15 | Hemorrhages, Intracerebral |
| 16 | Intracerebral Hemorrhages |
| 17 | Hemorrhage, Cerebral |
| 18 | Cerebral Hemorrhages |
| 19 | Hemorrhages, Cerebral |
| 20 | Brain Hemorrhage, Cerebral |
| 21 | Brain Hemorrhages, Cerebral |
| 22 | Cerebral Brain Hemorrhage |
| 22 | Cerebral Brain Hemorrhages |
| 23 | Hemorrhage, Cerebral Brain |
| 24 | Hemorrhages, Cerebral Brain |
| **25** | **1 OR 2-24** |
| 26 | Tranexamic Acid |
| 27 | AMCHA |
| 28 | trans-4-(Aminomethyl)cyclohexanecarboxylic Acid |
| 29 | t-AMCHA |
| 30 | AMCA |
| 31 | Anvitoff |
| 32 | Cyklokapron |
| 33 | Ugurol |
| 34 | KABI 2161 |
| 35 | Spotof |
| 36 | Transamin |
| 37 | Amchafibrin |
| 38 | Exacyl |
| **39** | **26 OR 27-38** |
| 40 | Randomized controlled trial |
| 41 | Controlled clinical trial |
| 42 | Randomized |
| 43 | Placebo |
| 44 | Clinical trials as topic |
| 45 | Randomly |
| 46 | Trial |
| **47** | **40-46** |
| **48** | **25 AND 39 AND 47** |
